# TWIK-1 contributes to the intrinsic excitability of dentate granule cells in mouse hippocampus

**DOI:** 10.1186/s13041-014-0080-z

**Published:** 2014-11-19

**Authors:** Oleg Yarishkin, Da Yong Lee, Eunju Kim, Chang-Hoon Cho, Jae Hyouk Choi, C Justin Lee, Eun Mi Hwang, Jae-Yong Park

**Affiliations:** Korea Institute of Science and Technology (KIST), Center for Functional Connectomics, Seoul, 136-791 Republic of Korea; Korea Research Institute of Bioscience and Biotechnology (KRIBB), Stem Cell Research Center, Daejeon, 305-806 Republic of Korea; School of Biosystem and Biomedical Science, College of Health Science, Korea University, Seoul, 136-703 Republic of Korea; Neuroscience Program, University of Science and Technology (UST), Daejeon, 305-350 Republic of Korea

**Keywords:** K2P channel, TWIK-1, Intrinsic excitability, Dentate gyrus granule cell

## Abstract

**Background:**

Two-pore domain K^+^ (K2P) channels have been shown to modulate neuronal excitability. However, physiological function of TWIK-1, the first identified member of the mammalian K2P channel family, in neuronal cells is largely unknown.

**Results:**

We found that TWIK-1 proteins were expressed and localized mainly in the soma and proximal dendrites of dentate gyrus granule cells (DGGCs) rather than in distal dendrites or mossy fibers. Gene silencing demonstrates that the outwardly rectifying K^+^ current density was reduced in TWIK-1-deficient granule cells. TWIK-1 deficiency caused a depolarizing shift in the resting membrane potential (RMP) of DGGCs and enhanced their firing rate in response to depolarizing current injections. Through perforant path stimulation, TWIK-1-deficient granule cells showed altered signal input-output properties with larger EPSP amplitude values and increased spiking compared to control DGGCs. In addition, supra-maximal perforant path stimulation evoked a graded burst discharge in 44% of TWIK-1-deficient cells, which implies impairment of EPSP-spike coupling.

**Conclusions:**

These results showed that TWIK-1 is functionally expressed in DGGCs and contributes to the intrinsic excitability of these cells. The TWIK-1 channel is involved in establishing the RMP of DGGCs; it attenuates sub-threshold depolarization of the cells during neuronal activity, and contributes to EPSP-spike coupling in perforant path-to-granule cell synaptic transmission.

**Electronic supplementary material:**

The online version of this article (doi:10.1186/s13041-014-0080-z) contains supplementary material, which is available to authorized users.

## Background

Two-pore domain K^+^ (K2P) channels are major contributors to background potassium conductance in cells and control resting membrane potential (RMP) as well as cellular excitability [1]. These K2P channels are modulated by a large variety of physical and chemical stimuli including temperature, membrane stretch, pH, polyunsaturated fatty acids, hormones and neurotransmitters. Different K2P channel family members have varying functions, with roles in adrenal gland development, thermal and mechanical nociception, and sensitivity to volatile-anesthetics [1]. Of the 15 isoforms in the K2P channel family, K2P1 (TWIK-1; **T**andem of pore domains in a **W**eak **I**nward rectifying **K**^+^ channel), was first cloned from human kidney [2]. Due to the low or non-existent functional expression of TWIK-1 in heterologous expression systems [3], TWIK-1 has long been considered to be non-functional as a plasma membrane channel.

However, and of interest to note, TWIK-1-deficient mice show defects in phosphate transport in the proximal tubule of kidney, as well as deviations in the RMP of pancreatic β cells [4,5]. It has also been recently reported that TWIK-1 is involved in the inward leak Na^+^ currents during pathological hypokalemia in cardiomyocytes [6]. In addition, TWIK-1 is activated by serotonin in entorhinal cortex stellate cells of the brain, as seen with pharmacological methods [7] and TWIK-1/TASK-3 heterodimeric channels mediate a halothane response in cerebellum granule cells [8]. We have also recently demonstrated that TWIK-1/TREK-1 heterodimers mediate a passive conductance in astrocytes [9]. These observations strongly suggest about important physiological roles for TWIK-1 channels, although the electrophysiological properties and functional significance of TWIK-1 are not yet fully understood.

The dentate gyrus (DG), sitting between the entorhinal cortex and the CA3 area, is the main gateway to the hippocampus. DG principal neurons, the dentate gyrus granule cells (DGGCs), are capable of effectively blocking or filtering excitatory input from the entorhinal cortex and controlling the amount of excitation that gets through to the hippocampus [10]. The filtering properties of these cells are indispensable for hippocampal-dependent memory processes [11]. DGGCs rely on strong GABA-mediated inhibition and relatively low intrinsic excitability [12-15]. As mRNAs of TWIK-1 have been known to be expressed in DGGCs [16,17], we hypothesized that TWIK-1 may contribute to the electrical properties of DGGCs.

The present study takes advantage of the TWIK-1 gene silencing technique by stereotaxic delivery of adenovirus-carried TWIK-1 shRNA to dentate gyrus, as well as brain slice electrophysiology. Using these methods, we have found that TWIK-1 contributes to the outwardly rectifying potassium currents in DGGCs and determines the intrinsic electrical membrane properties of the cells in brain slices.

## Results

### TWIK-1 is expressed in dentate granule cells of mouse hippocampus

**Figure 1:**
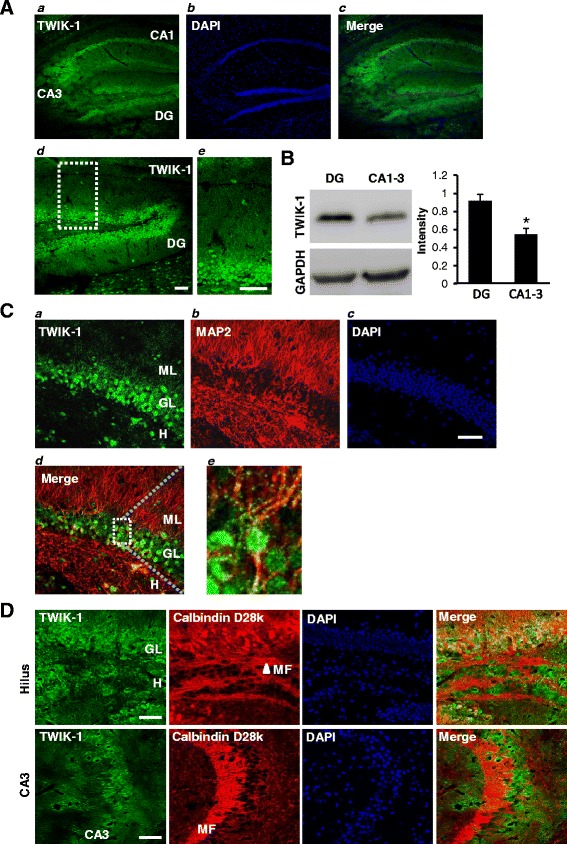
**TWIK-1 is expressed in mouse hippocampal dentate granule cells. (A)** Representative fluorescence immunostaining images show that TWIK-1 **(** ***a*** **)** is highly expressed in dentate granular layer and CA1-3 regions. DAPI staining **(** ***b*** **)** indicates the overall hippocampal sub-regions including dentate gyrus and CA1-3. Merged image **(** ***c*** **)** demonstrates co-localization of TWIK-1 with principal cells in all hippocampal sub-regions.*** (d)*** Magnified image of dentate gyrus, showing co-localization of TWIK-1 with dentate granule cells. ***(e)*** Magnified image of the dotted area indicated in ***(d)***. **(B)** Representative Western Blot data for the expression of TWIK-1 in dentate gyrus and CA1-3 region of the hippocampus (N = 3 mice, P < 0.01, Student’s unpaired *t*-test). **(C)** Representative immunostaining images with TWIK-1 ***(a)***, MAP2 ***(b)***, and DAPI ***(c)***. ML, molecular layer; GL, granule layer; H. hilus. Merged TWIK-1 and MAP2 staining image ***(d)*** showing that TWIK-1 is co-localized with MAP2 in dendrites of dentate granule cells. High magnification image ***(e)*** of dotted rectangle in (***d***) shows that MAP2-positive proximal dendrites of granule layer cells are co-localized with TWIK-1. Note the presence of TWIK-1 positive cells in the molecular layer (ML) of the dentate gyrus and the hilus (H). **(D)** Double immunostaining with TWIK-1 (green) and calbindin D28k (red) demonstrates that TWIK-1 is only co-localized with calbindin D28k in the granule layer (GL) but not in the hilus (H) or CA3. DAPI stains neuronal cells in the granule cell layer and CA3 layer. Scale bar, 50 μm. DG: dentate gyrus, GL: granule layer, CA1: cornu ammonis 1, CA3: cornu ammonis 3, ML: dentate molecular layer, H: dentate hilus, MF: mossy fibers.

TWIK-1 mRNAs are reportedly expressed in DGGCs [16,17]. However, the physiological function of TWIK-1 in these cells has never been examined. To determine TWIK-1 expression and the subcellular localization of this protein in DGGCs, we performed immunohistochemistry with an anti-TWIK-1 antibody in mouse brain slices. The specificity of the antibody against TWIK-1 has been determined previously [9] and also confirmed by immunohistochemistry analysis using mouse kidney and mouse skeletal muscle tissues (as a positive and a negative control for TWIK-1 expression, respectively: Additional file 1: Figure S1). We found that TWIK-1 channels are broadly expressed in the hippocampus, including DG and CA1-3 regions (Figure 1A). The expression of TWIK-1 in hippocampus was further confirmed by Western Blot analysis (Figure 1B) showing a relatively higher expression of TWIK-1 in DG compared to that in CA1-3 regions. In the DG, TWIK-1 expression was detected mainly in the DGGCs. Some sparsely distributed TWIK-1 positive cells were also present in the molecular layer and in the hilus (Figure 1C). We further determined the subcellular localization of TWIK-1 in DGGCs by co-staining with antibodies against TWIK-1 and calbindin D29k (a marker for dentate granule cells together with their mossy fiber projections) or MAP2 (dendritic marker). TWIK-1 was found to co-localize with MAP2 in some proximal dendrites of granule layer cells (Figure 1C) but not with calbindin D29k in mossy fibers in the hilus or CA3 regions (Figure 1D). Collectively, these data show that TWIK-1 is clearly expressed in the soma and noticeably in the proximal dendrites, but not in the distal dendrites and axons of DGGCs.

### TWIK-1 mediates outwardly rectifying K^+^ currents in dentate granule cells

Expression of TWIK-1 channels in DGGCs strongly suggests that TWIK-1 might contribute to the electrical properties of these cells. To investigate such a possible role for TWIK-1 in DGGCs, we first examined the effect of knocking down TWIK-1 on the whole-cell currents in these cells. For this purpose we constructed an adenovirus (Ad) carrying a small hairpin-forming interference RNA (shRNA) targeted against mouse TWIK-1. This virus also contained DNA encoding a fluorescent marker, mCherry, which permitted visualization of the location and amount of viral injection. The specificity of the TWIK-1 shRNA has been documented previously [9].

**Figure 2:**
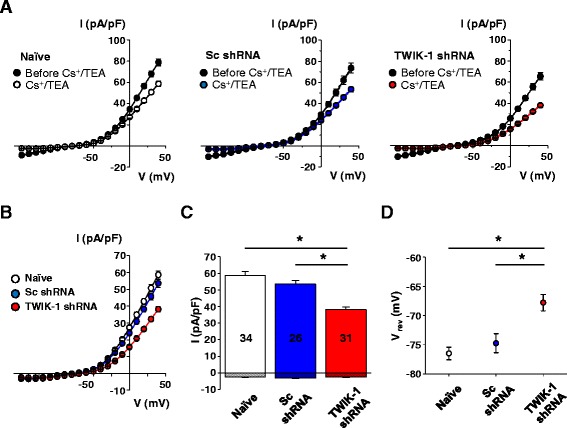
**TWIK-1 contributes to outwardly rectifying currents in dentate granule cells. (A)** Averaged current-voltage (*I-V*) relationships of whole-cell currents in dentate granule cells measured in standard ACSF and after subsequent application of Cs^+^/TEA. **(B)** Current-voltage relationship of the whole-cell currents in naïve (left panel; n = 34 cells, N = 3 mice), Sc shRNA (middle panel; n = 26 cells, N = 3 mice) or TWIK-1 shRNA (right panel; n = 31 cells, N = 3 mice) expressing dentate granule cells in the presence of Cs^+^/TEA. Whole-cell currents were elicited by 1 s duration ramp pulses descending from 40 mV to -150 mV from a holding potential of -70 mV. **(C)** Summary bar charts for **(B)**. Shown are mean values of whole-cell current density in naïve, Sc shRNA or TWIK-1 shRNA expressing granule cells measured after subsequent application of the blockers; current density values are depicted at -150 mV *(shadowed bars below baseline)* and 40 mV *(open bars)*. **(D)** Reversal potential values of the whole-cell currents in naïve (n = 34 cells, N = 3 mice), Sc shRNA (n = 26 cells, N = 3 mice) or TWIK-1 shRNA (n = 31 cells, N = 3 mice) expressing dentate granule cells. All data are represented as mean ± S.E.M; *P < 0.05, Student’s unpaired *t*-test.

DGGCs are known to functionally express several types of K^+^ channels, including the inwardly rectifying (K_ir_) and voltage-gated (K_*v*_) potassium channels [18-22]. Therefore, to measure TWIK-1-mediated currents in DGGCs, we applied a mixture of the commonly-used K_ir_ channel blocker, Cs^+^ (1 mM) and K_*v*_ channel blocker, TEA (2 mM). We shall refer to this mixture as Cs^+^/TEA. In standard ACSF, the whole-cell current-voltage (*I-V*) relationship of DGGCs showed prominent inward and outward currents. As shown in Figure 2A, administration of Cs^+^/TEA caused a marked reduction of both outward and inward currents (by 25.7 ± 3.04% and 75.87 ± 3.48% at 40 mV and -150 mV, respectively). Notably, Cs^+^/TEA almost completely abolished the inwardly rectifying component of the *I-V* curve, while the outwardly-rectifying component was also seen to be reduced. Remaining Cs^+^/TEA-resistant currents in naïve DGGCs had a prominent outwardly rectifying *I-V* relationship with a current density of -2.4 ± 0.3 pA/pF at -150 mV and 58.6 ± 2.4 pA/pF at 40 mV. TWIK-1 shRNA significantly reduced only outward currents (-2.5 ± 0.2 pA/pF at -150 mV and 38.1 ± 1.7 pA/pF at 40 mV), while the Scrambled shRNA (Sc shRNA) control did not affect the *I-V* relationship (-3.1 ± 0.4 pA/pF at -150 mV and 53.5 ± 2.3 pA/pF at 40 mV: Figures 2B, C). The reversal potential of the currents in TWIK-1-deficient granule cells was shifted towards a positive voltage range (-67.8 ± 1.4 mV) compared to that in naïve or Scrambled control cells (-76.5 ± 1.1 mV and -74.7 ± 1.6 mV, respectively: Figure 2D), implying a lack of potassium conductance in TWIK-1-deficient cells. Taken together, these results indicate that TWIK-1 contributes to electrical properties of the DGGC plasma membrane, behaving as an outwardly-rectifying K^+^ channel in DGGCs.

### Knockdown of TWIK-1 enhances intrinsic excitability of DGGCs

**Figure 3:**
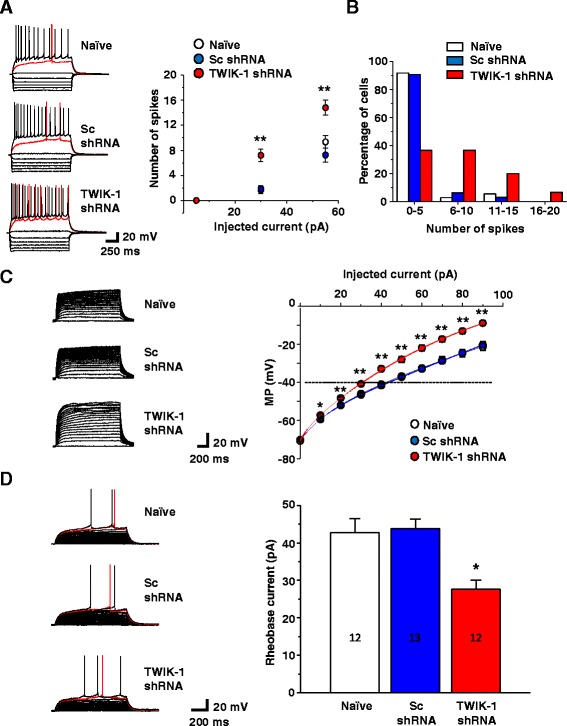
**TWIK-1 deficiency causes enhanced intrinsic excitability of dentate granule cells. (A)** Representative response of membrane potential to stepwise current injections (left panel). The current was injected into cells in 25 pA steps starting from -120 pA and up to 55 pA (1.2 sec step duration). Input-output properties of naïve (n = 36 cells), Sc shRNA (n = 32 cells) or TWIK-1 shRNA (n = 30 cells) expressing granule cells measured as the number of spikes *vs.* injected current intensity (right panel). **(B)** Distribution of cells according to excitability patterns. Plotted are percentage of cells with binned number of spikes fired during a 30 pA injected current step. **(C)** Representative response of membrane potential to stepwise current injections (left panel). Averaged response of membrane potential to stepwise current injection in naïve (n = 27 cells), Sc shRNA (n = 20 cells) or TWIK-1 shRNA (n = 21 cells) expressing cells (right panel). The RMP of cells was maintained at -70 mV. Current injection into the cell body was performed stepwise from -30 pA to 90 pA, in 5 pA steps. The solid lines are an exponential fit of the data plots. Dotted line indicates the spiking threshold level. **(D)** Representative traces of rheobase current measurements (left panel). The RMP of cells was kept at -70 mV and then depolarizing current was injected stepwise, in 2 pA steps until the membrane potential reached the firing threshold *(red traces)*. Averaged values of rheobase currents in naïve (n = 12 cells), Sc shRNA (n = 13 cells) or TWIK-1 shRNA (n = 12 cells) expressing cells (right panel). All data are represented as mean ± S.E.M; *P < 0.05, **P < 0.01 Student’s unpaired *t*-test.

Discovering that TWIK-1 knockdown brought about a positive shift in reversal potential in dentate granule cells, we next examined the effect of TWIK-1 shRNA on the intrinsic excitability of these cells in brain slices. The firing rates of dentate granule cells were determined as a function of injected currents. The firing rates in TWIK-1 shRNA-infected cells were higher than those in naïve or Sc shRNA-infected cells (Figures 3A, B). The characteristics of averaged action potentials such as the potential threshold or the amplitude of the action potentials in TWIK-1 shRNA-infected cells were not statistically different from such characteristics in naïve or Scrambled control cells. However, the RMP of TWIK-1-deficient DGGCs was significantly depolarized (-70.6 ± 0.6 mV), compared to both naïve (-74.6 ± 0.9 mV) and Scrambled control cells (-77.3 ± 1.2 mV: Additional file 2: Table S1).

The enhanced firing rate of TWIK-1-deficient DGGCs might be caused solely by the depolarized RMP, which reduces the voltage threshold for excitation to reach the threshold level of firing. In addition, and contributing to the establishment of RMP, K2P channel activity can attenuate the excitatory drive to neurons by providing a shunting potassium conductance, an effect that is strengthened with membrane depolarization [23,24]. Given the significant TWIK-1 shRNA-sensitive component in the whole-cell currents of DGGCs, we suggested that there is a marked shunting effect of TWIK-1 or the TWIK-1 containing channel-mediated conductance on excitatory drive in these cells. To address this possibility, we measured membrane depolarization in response to injected currents in TWIK-1-deficient DGGCs under controlled RMP conditions, in which RMP was maintained at -70 mV by constant current injection. The *I-V* relationship of TWIK-1-deficient DGGCs displays a less prominent outward rectification compared to the *I-V* of naïve or Sc shRNA-infected cells, evidence of a lack of shunting effect in TWIK-1-deficient DGGCs (Figure 3C). To further prove that a lack of TWIK-1-mediated shunting effect may influence the DGGC firing rate, we measured the rheobase current in TWIK-1-deficient DGGCs. Again, the RMP of cells was kept at -70 mV by constant current injection into the cell body. A depolarizing current of 2 pA was then injected stepwise until the membrane potential reached the threshold potential level at which a single spike was generated. The rheobase current was significantly smaller in TWIK-1-deficient DGGCs compared to that in naïve and Scrambled control cells (27.6 ± 2.5 pA, 42.7 ± 3.8 pA, 43.8 ± 2.6 pA, respectively; P < 0.05: Figure 3D), confirming that depolarization of the RMP of TWIK-1-deficient cells is not the sole cause of their enhanced firing rate, but that a lack of shunting during excitatory post synaptic potentials (EPSPs) also takes place.

These data provide evidence that TWIK-1 contributes to the intrinsic excitability of DGGCs by establishment of the RMP and by providing a potassium conductance, which attenuates membrane depolarization in response to excitatory current injection.

### Synaptic response of TWIK-1-deficient dentate granule cells

**Figure 4:**
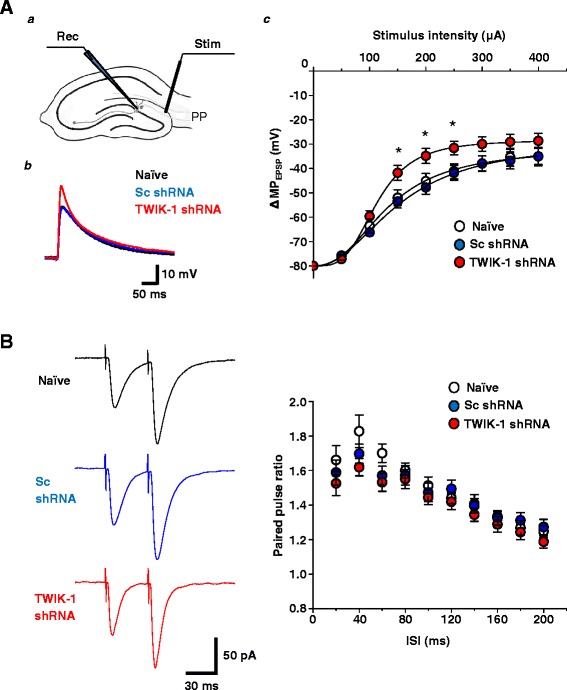
**TWIK-1 deficiency in dentate granule cells causes enhanced amplitude of evoked EPSPs in response to perforant path stimulation. (A) (** ***a*** **)** Schematic diagram for synaptically evoked responsiveness of dentate granule cells. PP: perforant path, Stim: stimulation, Rec: recording. **(** ***b*** **)** Representative traces of evoked EPSPs in naïve, Sc shRNA or TWIK-1 shRNA expressing dentate granule cells. Synaptic responses evoked by 150 μA stimulation of perforant path are shown. **(** ***c*** **)** Input-output properties of dentate granule cells. Plotted values are of evoked EPSP amplitudes in naïve (n = 13cells, N = 3 mice), Sc shRNA (n = 14 cells, N = 3 mice) or TWIK-1 shRNA (n = 12 cells, N = 3 mice) expressing granule cells. The RMP was maintained at -80 mV by steady current injection. For each stimulation intensity, ten perforant path stimuli were applied and the evoked EPSP responses were averaged. The solid line is a logistic fit of the data plots. ACSF contained 10 μM bicuculline and 10 μM CGP 55845; pipette solution contained 5 mM QX-314. (**B**) Representative traces for paired-pulse ratio measurements (left panel). Paired-pulse ratios in perforant path to granule cell synaptic transmission as a function of interstimulus interval (ISI) in naïve (n = 16 cells, N = 3 mice), Sc shRNA (n = 16 cells, N = 3 mice) or TWIK-1 shRNA (n = 16 cells, N = 3 mice) expressing cells (right panel). ACSF contained 10 μM bicuculline and 10 μM CGP 55845; pipette solution contained 5 mM QX-314. All data are represented as mean ± S.E.M; *P < 0.05, Student’s unpaired *t*-test.

A reduced leak potassium conductance may cause a stronger synaptically-evoked depolarization of the plasma membrane of TWIK-1 DGGCs. To test whether a TWIK-1 shunting effect might influence synaptically-evoked membrane events, we estimated the amplitude, rise and decay kinetics of evoked excitatory post synaptic potentials (eEPSPs) in TWIK-1-deficient DGGCs. The RMP was maintained at -80 mV by constant current injection. Comparative analysis of eEPSP amplitudes showed a significant difference between TWIK-1-deficient granule cells and naïve or Scrambled control cells over a stimulus intensity range of 150 - 250 μA, with larger values for EPSP amplitude being observed in TWIK-1-deficient cells (Figure 4A). The average rise time of eEPSPs was not statistically different among TWIK-1 shRNA, Sc shRNA-infected or naïve DGGCs within a stimulation intensity range of 50 - 400 μA (data not shown). At the same time, unaltered paired-pulse ratios calculated from a series of paired-pulse interval stimulations of TWIK-1-deficient DGGCs, rule out any alteration in release probability at perforant path terminals after TWIK-1 knockdown in DG (Figure 4B). Thus, the altered eEPSP properties in TWIK-1-deficient DGGCs can be explained by changes in the properties of post-synaptic cells.

**Figure 5:**
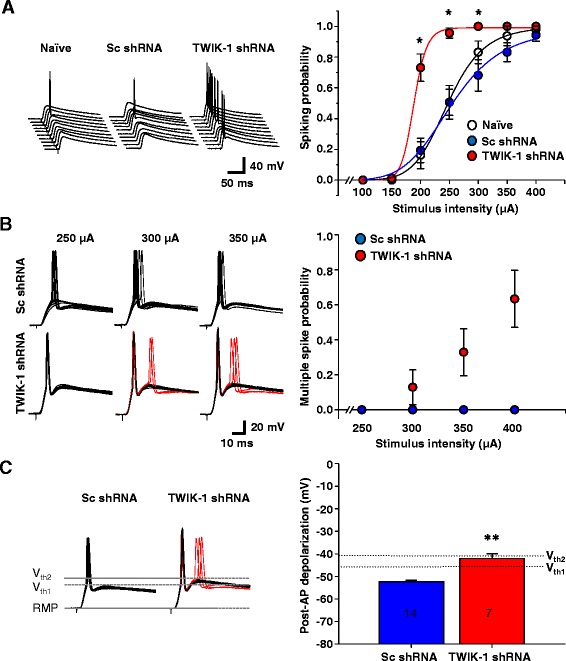
**TWIK-1 knockdown in dentate granule cells affects signal input-output properties of the cells. (A)** Representative traces from spiking probability measurements (left panel). Synaptic responses evoked by 200 μA stimulation of perforant path are shown. Input-output properties of dentate granule cells (right panel). Plotted are the values of spiking probability in naïve (n = 16 cells, N = 3 mice), Sc shRNA (n = 16 cells, N = 3 mice) or TWIK-1 shRNA (n = 16 cells, N = 3 mice) expressing dentate granule cells. The solid line is a logistic fit of the data plots. **(B)** Representative traces from spiking probability measurements (left panel). Traces with multiple spikes are shown in red. Averaged input-output properties of the subpopulation of the burst discharging cells (right panel). Plotted are the averaged values of probability of firing more than one spike per eEPSP in TWIK-1 shRNA or Sc shRNA expressing cells. **(C)** Representative traces of ten evoked EPSP responses in Sc shRNA and TWIK-1 shRNA expressing dentate granule cells (stimulation intensity was 350 μA) (left panel). The dotted lines indicate RMP, the voltage threshold for the first AP (V_th1_) and the voltage threshold for the second AP (V_th2_) in the burst response. Traces containing the second spike are shown in red. Summary bar graph for averaged depolarization level after the termination of the first AP in Sc shRNA and a population of TWIK-1 shRNA expressing cells with multiple spikes (right panel). The dotted lines show the threshold levels for the first (V_th1_) and the second (V_th2_) AP during a single EPSP. Number of cells are shown within the bars. All data are represented as mean ± S.E.M; *P < 0.05, **P < 0.01 Student’s unpaired *t*-test.

The enhanced intrinsic excitability of TWIK-1-deficient DGGCs may lead to an altered efficiency of signal transmission through these cells. To address this possibility, we estimated the spiking probability of TWIK-1-deficient DGGCs as a function of perforant path stimulation intensity. The threshold for synaptically-evoked action potentials was somewhat more hyperpolarized compared to that evoked by somatic current injection (48.3 ± 1.4 mV; n = 18, 46.5 ± 1.6 mV; n = 18, and 46.1 ± 1.4 mV; n = 21 in naïve, Scrambled, and TWIK-1-deficient cells, respectively). We found that the average spiking probability in TWIK-1-deficient granule cells reached its maximum at lower stimulus intensity values compared to those in naïve and Scrambled control cells (Figure 5A). In addition, in 7 out of 16 TWIK-1-deficient cells, a single pulse of supra-maximal intensity evoked a burst firing response (2 or 3 action potentials), while only one action potential was evoked by single supra-maximal intensity stimulation in naïve and Sc shRNA-infected cells (Figures 5B, C). The threshold for the second spike in the burst was depolarized by 7.47 ± 0.98 mV relative to the first spike (n = 7, P < 0.01, paired Student’s *t-*test). The probability of a second spike occurring was increased with increasing stimulation intensity (Figure 5B). After termination of the action potential evoked by supra-threshold stimulation, the membrane potential in the control Sc shRNA-infected cells is of sub-threshold depolarization levels (Figure 5C), while in a burst-firing fraction of TWIK-1-deficient cells, the membrane potential was depolarized above the first spike threshold level and it reached the threshold for the second spike. However, the mechanism of multiple spiking in TWIK-1-deficient DGGCs requires more in-depth study. Overall, we conclude that TWIK-1 or the TWIK-1 containing channel activity contributes to signal input-output properties in DGGCs.

## Discussion

Our data demonstrate that TWIK-1 is expressed in DGGCs and localized mainly in the cell soma. TWIK-1-deficient GCs had an attenuated outwardly rectifying current density with a reversal potential for currents that was more depolarized than that seen in naïve or Sc shRNA expressing cells. These data show that TWIK-1 contributes to the outwardly rectifying potassium current in DGGCs. The expression of outwardly rectifying leak potassium channels in neuronal cells suggests they contribute to excitability in these cells by establishing the RMP [25-27] or by shunting excitation during neuronal activity [23,24]. The shunting effects of K2P channels can result from an increased input from the potassium conductance associated with K2P channel opening, which reduces membrane excitability by attenuating excitatory drive.

The shunting influence of inwardly rectifying currents on the excitability of neuronal cells, particularly, dentate granule cells, was recently demonstrated [28]. The contribution of outwardly rectifying potassium channels to somatic shunting was described in detail for the K_*v*_3 voltage-gated potassium channel in starburst amacrine cells [29]. In these neurons, activation of K_*v*_3 channels provides a voltage-dependent shunt that limits depolarization of the soma to potentials more positive than -20 mV, the voltage threshold of the K_*v*_3 channel. Unlike voltage-gated potassium channels, K2P channels do not have an activation threshold. They may therefore provide shunting over the whole physiological range of membrane potentials. We found that TWIK-1-deficient granule cells showed a small but significant depolarizing shift in RMP. In addition, the data we obtained in voltage-clamp experiments with TWIK-1-deficient DGGCs, suggests a marked activity of TWIK-1-mediated current at a membrane potential of ≈ -40 mV, the spiking threshold level in DGGCs cells. Considering that in a voltage range from the RMP to the spike threshold potential level, the contribution of TWIK-1 channels to the electrical properties of the DGGC plasma membrane is relatively high, we assumed that as a result of this activity in sub-threshold membrane events, an attenuation in the amplitude of membrane depolarization occurs. Indeed, in current-clamp experiments, the relationship between membrane voltage and injected current in TWIK-1-deficient, naïve or Sc shRNA-infected DGGCs revealed a significantly outward rectification in TWIK-1-deficient cells, suggesting that the TWIK-1 current attenuates depolarization of DGGCs in response to current injection.

The influence of TWIK-1-mediated shunting on the excitability of DGGCs was confirmed by measurement of rheobase currents, which were smaller in TWIK-1-deficient cells compared to those in naïve or Scrambled control cells, under the same RMP conditions. A lack of TWIK-1-mediated shunting was also observed in synaptically-evoked depolarizations of the plasma membrane. Under constant RMP conditions, eEPSPs in TWIK-1-deficient cells were of larger amplitude compared to those in naïve and Scrambled control cells, showing that the TWIK-1-mediated conductance attenuates eEPSP amplitude. At the same time, the release probability in perforant path terminals was unaltered, confirming post-synaptic mechanisms for the observed changes in synaptic response. Consistent with a higher intrinsic excitability, TWIK-1-deficient DGGCs had altered excitatory postsynaptic potential-spike coupling, which was demonstrated by an enhanced spiking probability in these cells in response to perforant path stimulation. Of interest to note, is that the DGGC mode of firing, which normally involves the generation of a single spike per one supra-threshold eEPSP event, was converted to a graded burst discharge with increasing stimulus intensity in 44% of TWIK-1-deficient cells. This multiple spike phenomenon could be explained by larger eEPSPs in a fraction of TWIK-1-deficient granule cells that allow membrane potential to be depolarized up to the value of the second spike threshold level after termination of the first action potential. Overall, the combined effect of a depolarized RMP and a lack of shunt in TWIK-1-deficient DGGCs results in the altered signal input-output properties in these cells with increased spike probability and altered EPSP-spike coupling.

Altered input-output properties of TWIK-1-deficient DGGCs, especially changes in firing mode in a sub-population of TWIK-1 knockdown cells, reveal an important role for TWIK-1 in the filtering properties of DGGCs and hence the physiological function of DG as a gateway to the hippocampus. Though the precise regulatory mechanisms of TWIK-1 channel activity are not well established, we speculate that modulation of the intrinsic excitability of DGGCs by up- or down-regulation of TWIK-1 channel activity may occur either in normal or pathological states that might affect the filtering properties of DGGCs and hence influence information processing from entorhinal cortex to hippocampus.

The outwardly rectifying properties of TWIK-1-mediated currents seen in DGGCs was a somewhat surprising finding as currents mediated by TWIK-1 expressed heterologously in *Xenopus laevis* oocyte have been characterized as weakly inwardly rectifying [2,30]. The reason for the observed discrepancy between the biophysical characteristics of TWIK-1 currents in *Xenopus laevis* oocytes and our own data is not clear for now. However, it has been suggested that for its functional expression [31], TWIK-1 might require cellular factors that have yet to be discovered. These may regulate the surface expression of TWIK-1 or its channel properties. Worth noting, is that biophysical properties of the deSUMOylated TWIK-1 current were described to be outwardly rectifying [3,32]. Thus, the properties of cloned TWIK-1 might differ from those of TWIK-1 in its native environment. As well as this, recent publications provide evidence for heteromerization between TWIK-1 and other K2P channels, such as TASK-3 and TREK-1. Heteromerization between TWIK-1 and TASK-3 results in formation of a channel with outwardly rectifying properties [8], while TWIK-1/TREK-1 heterodimer-mediated currents demonstrate a linear *I-V* relationship [9]. It requires further in-depth investigations what is the molecular mechanisms that confers TWIK-1 with a functional channel properties in DG GCs.

## Conclusions

The physiological function of TWIK-1, the first identified member of the K2P channel family, in neuronal cells is largely unknown. We demonstrate, for the first time, a functional expression of TWIK-1 in DGGCs in mouse hippocampus. TWIK-1 mediates outwardly rectifying currents and is involved in establishing the RMP of DGGCs. TWIK-1 attenuates sub-threshold depolarization of the cells during neuronal activity, and contributes to EPSP-spike coupling in perforant path-to-granule cell synaptic transmission. Taken together, these results clearly showed that TWIK-1 has a pivotal role in the electrical properties of DGGCs.

## Methods

### Animals

Male C57BL/6 mice, aged 7-8 weeks old, were used for all experiments. Animal care and handling were performed according to instructional guidelines of the Korea Institute of Science and Technology (Seoul, Korea).

### Construction of the recombinant adenovirus vector

pSicoR-Scrambled and pSicoR-TWIK-1 shRNA plasmid [9] were cloned into pDONR™ 207 vectors (Invitrogen), and confirmed by DNA sequencing. An LR recombination reaction was performed between the cloned plasmid and pAd/CMV/V5-DEST™ destination vector, using LR Clonase™ enzyme mix. Adenoviral vectors carrying each shRNA were then linearized by PacI digestion and used to transfect 293A cells using Lipofectamine® 2000 reagent. The viral titer was determined in a 96-well plate according to the manufacturer’s instructions.

### Stereotaxic virus injections

Virus injections were performed on deeply anesthetized mice of 7 weeks old, which were placed in a stereotaxic frame (Kopf Instruments). Anesthesia was induced with Avertin® (2,2,2-tribromethanol in 2-methyl-2-butanol) via intraperitoneal injection. Briefly, the scalp was opened and two holes were drilled in the skull (-2.0 mm AP, ±1.3 mm ML from bregma). Ad-shTWIK-1-mCherry or Ad-Scrambled-mCherry (2 μl per side) was injected bilaterally into the dentate gyrus (1.6 mm DV from the dura) through a glass microdispenser (VWR, USA) with a syringe pump (KD Scientific, USA) that infused the virus at a speed of 0.2 μl/min. The microdispenser was left in place for 2 min before and 2 min after the injection.

### Fluorescence immunohistochemistry and image collection

Mouse tissues were obtained following intracardiac perfusion with saline and 4% paraformaldehyde solution and 20 μm-thick frozen tissue sections were prepared using a cryostat. Sections were mounted onto slides and permeabilized with 0.5% Triton X-100 in PBS for 20 min at room temperature (RT, 20-22 °C) followed by blocking with 10% donkey serum and 0.1% Triton X-100 in PBS for 1 hr at RT. Tissues were incubated overnight with primary antibodies, such as rabbit anti-TWIK-1 polyclonal antibody (1:100; Alomone, Jerusalem, Israel) or chicken anti-MAP2 polyclonal antibody (1:1000; Abcam, Cambridge, MA), at 4°C. For detection, suitable fluorescence (Alexa fluor)-tagged secondary antibodies (Molecular Probes, Eugene, OR) were used and tissues were counterstained with DAPI.

### Western blotting

The mouse brain was isolated and the DG and other hippocampal regions (CA) were dissected out. The obtained DG and CA tissues were lysed with radioimmunoprecipitation assay buffer and processed for Western Blotting using rabbit anti-TWIK-1 antibody (1:1000; Abcam, Cambridge, MA) and mouse anti-GAPDH (1:5000; Abcam, Cambridge, MA). Signals were detected by enhanced chemiluminescence (GE Healthcare, UK) following probing with the appropriate HRP-conjugated secondary antibodies (Jackson Laboratory, Bar Harbor, ME). Each experiment was performed with samples from three independent groups. Signal intensity was quantified using ImageJ software.

### Brain slice preparation and electrophysiology

Brain slice electrophysiology was performed at 6-7th day after virus injections. Mice were decapitated after induction of anesthesia by ether. Mouse brains were rapidly removed and placed into ice-cold modified artificial cerebrospinal fluid (ACSF) containing (in mM) 130 NaCl, 2.5 KCl, 1.25 KH_2_PO_4_, 3.0 MgCl_2_, 1.0 CaCl_2_, 26 NaHCO_3_, and 10.0 D-glucose. For electrophysiological experiments 400 μm thick horizontal sections containing hippocampi were prepared using a vibratome (Linear slicer DSK, Japan) in ice-cold oxygenated ACSF. After resting for 1-2 h in ACSF of the same composition, at RT, slices were placed onto the recording chamber and experiments were performed in ACSF that contained (in mM): 130 NaCl, 2.5 KCl, 1.25 KH_2_PO_4_, 1.5 MgCl_2_, 1.5 CaCl_2_, 26 NaHCO_3_, and 10.0 D-glucose. Hippocampal slices were visualized with an Olympus BX51WI microscope equipped with epifluorescence. mCherry-positive cells were selected for whole-cell patch recording. Patch pipettes were made from borosilicate glass and had a resistance of 7–10 MΩ. Pipette solution contained (in mM): 120 K-gluconate, 10 KCl, 1 MgCl_2_, 0.5 EGTA, 40 HEPES (pH adjusted to 7.2 with KOH). In voltage-clamp experiments the holding membrane potential was set to -70 mV. Series and input resistances were monitored throughout the experiment using a -5 mV pulse. Recordings were considered stable when both the series and input resistance, and the RMP did not change >20%. Recordings were filtered at 2 kHz and digitized at 10 kHz. In current-clamp experiments, currents were injected stepwise, in 25 pA steps. Step duration was 1.2 s. Granule cells were identified by morphological and physiological criteria such as a highly negative RMP and the absence of a voltage “sag” in response to hyperpolarizing current injection. Recordings were made from granule cells located in the middle or the outer third of the dentate granule cell layer. Granule cells were only included in the study if they had a RMP more negative than -60 mV and if the amplitude of the action potential was higher than 50 mV. In voltage-clamp experiments whole-cell currents were elicited by ramp pulses descending from 40 mV to -150 mV for 1 sec. The descending epoch in the protocol was preceded with a voltage step to 40 mV for 500 ms from the holding potential of -70 mV. Synaptic responses were evoked by 0.1 Hz stimulation of perforant path fibers (100 μsec duration; 100–1000 μA intensity) via a constant current isolation unit (WPI, USA) connected to a concentric electrode (FHS Inc, USA). Perforant path fibers were stimulated by placing the stimulation electrode in the outer or the middle third of the molecular layer of the dentate. Spiking probability was calculated as the ratio of a number of successful (spike-generating) stimulations to the total number of stimulations (10 stimulations delivered at 0.1Hz). The stimulus intensity was in the range of 100 to 400 μA, applied in 50 μA increments. Paired-pulses were applied with inter-pulse intervals ranging from 20 ms through 2000 ms, in 20 ms increments. All experiments were performed in the presence of GABA_A_ and GABA_B_ antagonists bicuculline (10 μM) and CGP 55845 (10 μM), respectively. When the response of membrane potential to stepwise current injections was examined, the RMP was maintained at -70 mV. ACSF solution contained 50 μM D-AP5, 10 μM CNQX, 10 μM bicuculline, 10 μM CGP 55845, 2 mM tetraethyl ammonium chloride, and 0.5 mM NiCl_2_; pipette solution contained 5 mM QX-314 to block the action potentials firing. The recordings for representative traces of rheobase current measurements were performed in ASCF containing 50 μM D-AP5, 10 μM CNQX, 10 μM bicuculline, and 10 μM CGP 55845. Data were collected with a MultiClamp 700B amplifier (Molecular Devices) using Clampex 10 data acquisition software (Molecular Devices) and were digitized with Digidata 1322A (Molecular Devices). All experiments were performed at RT.

### Chemicals

Bicuculline methobromide, CGP 55845 hydrochloride, D-AP5, CNQX, and QX-314 were purchased from Tocris. Other reagents were purchased from Sigma or from Calbiochem.

### Statistical analysis

Numerical data are presented as means ± S.E.M. Error bars in graphs denote the standard error of the mean. The statistical significance of data was assessed by Student’s unpaired or paired *t*-test. The significance level is shown with asterisks (*P < 0.05 or **P < 0.01).

## Additional files

Additional file 1: Figure S1. Validation of anti-TWIK-1 antibody for immunohistochemistry on frozen tissue. Representative fluorescence immunostainingimages show that TWIK-1 antibody detected positive signals in apical localization of proximal tubule of mouse kidney (A), but notin mouse skeletal muscle (B), in agreement with published studies [2,30]. Scale bar, 20 μm. Additional file 2: Table S1. Membrane electrical properties of dentate granule cells.
