# Supplementary material for: TWIK-1 contributes to the intrinsic excitability of dentate granule cells in mouse hippocampus
Source: Mol Brain. 2014 Nov 19;7:80. doi: 10.1186/s13041-014-0080-z (PMC4240835; doi:10.1186/s13041-014-0080-z)
Supplement: Additional file 1: Figure S1. — Validation of anti-TWIK-1 antibody for immunohistochemistry on frozen tissue. Representative fluorescence immunostainingimages show that TWIK-1 antibody detected positive signals in apical localization of proximal tubule of mouse kidney (A), but notin mouse skeletal muscle (B), in agreement with published studies [2,30]. Scale bar, 20 μm. [file 13041_2014_80_MOESM1_ESM.pdf]

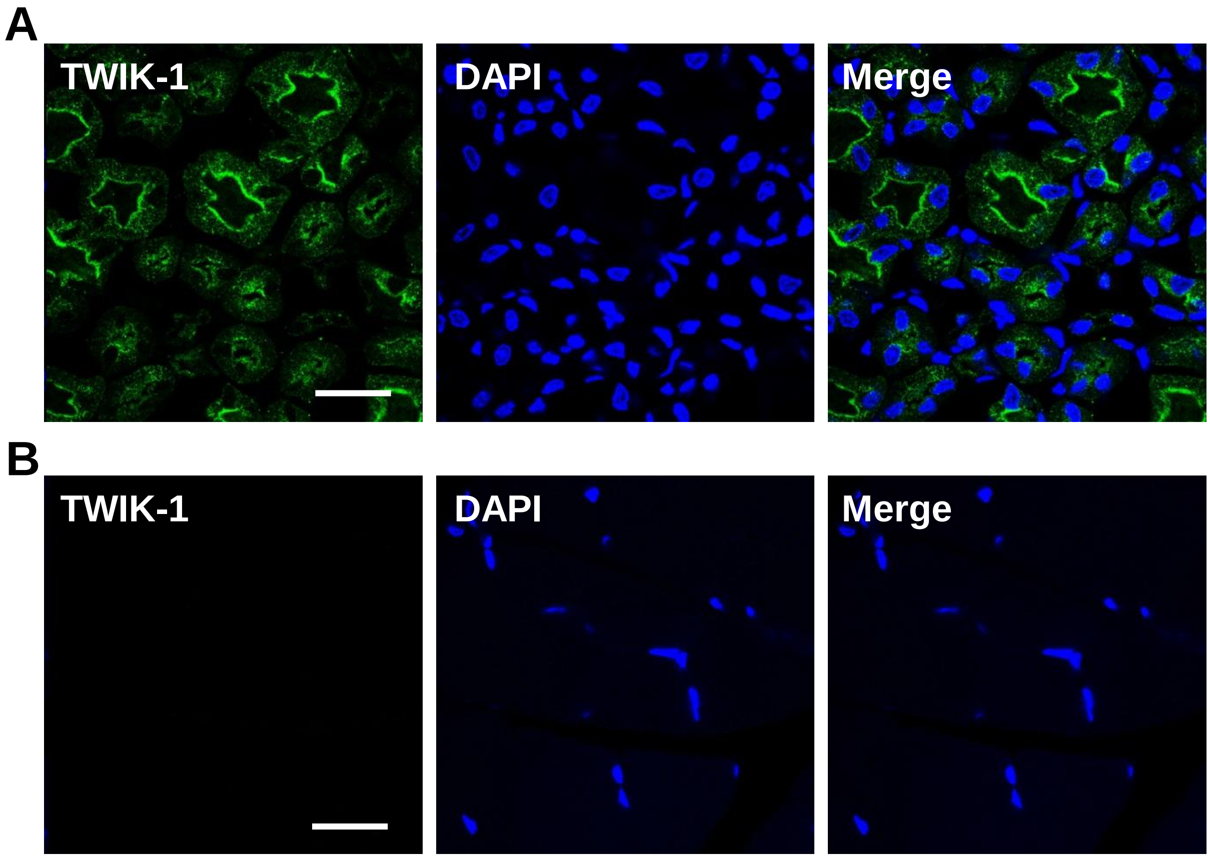

Supplementary Figure 1. Validation of anti-TWIK-1 antibody for immunohistochemistry on frozen tissue. Representative fluorescence immunostaining images show that TWIK-1 antibody detected positive signals in apical localization of proximal tubule of mouse kidney **(A)**, but not in mouse skeletal muscle **(B)**, in agreement with published studies [2,30]. Scale bar, 20 μm.
