# Supplementary material for: TWIK-1 contributes to the intrinsic excitability of dentate granule cells in mouse hippocampus
Source: Mol Brain. 2014 Nov 19;7:80. doi: 10.1186/s13041-014-0080-z (PMC4240835; doi:10.1186/s13041-014-0080-z)
Supplement: Additional file 2: Table S1. — Membrane electrical properties of dentate granule cells. [file 13041_2014_80_MOESM2_ESM.pdf]

Table S1. Membrane electrical properties of dentate granule cells

Membrane properties	Naïve (n=36, N=3)	Sc shRNA (n=32, N=3)	TWIK-1 shRNA (n=30, N=3)
Resting membrane potential (mV)	-74.9±0.6	-77.3±1.2	-70.6±0.6*
Input resistance (MΩ)	185.8±7.2	213.2±9.5	231.78±9.8
Firing threshold potential (mV)	-37.2±0.7	-38.3±1.0	-39.3±0.7
Number of spikes (at 30pA)	1.7±0.6	1.9±0.5	7.2±1.0*
AP amplitude (mV)	77.5±1.8	75.1±1.7	73.5±1.5

Data are expressed as mean ± S.E.M (naïve, n=36; Sc shRNA, n=32; TWIK-1 shRNA, n = 30).
P values were calculated using the Student's unpaired or paired *t*-test. * indicates $P < 0.05$.
